# Genetic Diversity in Bronchial Asthma Susceptibility: Exploring the Role of Vitamin D Receptor Gene Polymorphisms in Varied Geographic Contexts

**DOI:** 10.3390/ijms25031943

**Published:** 2024-02-05

**Authors:** Natalia Paramonova, Ilva Trapina, Brigita Gradauskiene (Sitkauskiene), Samanta Plavina, Laura Tamasauskiene, Daina Bastyte, Ingrida Rumba-Rozenfelde, Sandra Tapina, Ieva Stakaitiene, Rasa Ugenskiene, Lawrence Shih-Hsin Wu, Jiu-Yao Wang, Miao-Hsi Hsieh, Pei-Chi Chen, Nikolajs Sjakste

**Affiliations:** 1 Laboratory of Genomics and Bioinformatics, Institute of Biology, University of Latvia, LV-1004 Riga, Latvia; natalia.paramonova@lu.lv (N.P.); samanta.plavina@lu.lv (S.P.); nikolajs.sjakste@lu.lv (N.S.); 2 Department of Immunology and Allergology, Lithuanian University of Health Sciences, LT-50161 Kaunas, Lithuanialaura.tamasauskiene@lsmu.lt (L.T.); daina.bastyte@lsmu.lt (D.B.); 3 Faculty of Medicine, University of Latvia, LV-1586 Riga, Latvia; ingrida.rumba-rozenfelde@lu.lv (I.R.-R.); sandra.feierabende@gmail.com (S.T.); 4 Department of Genetics and Molecular Medicine, Lithuanian University of Health Sciences, LT-50161 Kaunas, Lithuania; ieva.golubickaite@lsmu.lt (I.S.); rasa.ugenskiene@lsmuni.lt (R.U.); 5 Graduate Institute of Biomedical Sciences, China Medical University, Taichung 406040, Taiwan; lshwu@hotmail.com; 6 Research Center of Allergy, Immunology, and Microbiome (AIM), China Medical University Hospital, Taichung 404327, Taiwan; a122@mail.ncku.edu.tw (J.-Y.W.); karinadrift@gmail.com (M.-H.H.); simple48686@gmail.com (P.-C.C.); 7 Department of Allergy and Immunology, China Medical University Children’s Hospital, Taichung 404327, Taiwan

**Keywords:** bronchial asthma, vitamin D receptor, vitamin D, polymorphisms, 25(OH)D level, TaqI, FokI, Bmsl, ApaI

## Abstract

Bronchial asthma (BA) exhibits varying prevalence across global populations, prompting a comprehensive investigation into genetic and environmental determinants. Vitamin D is a potent immunomodulator capable of suppressing inflammatory signals in several cell types involved in the asthmatic response; it exerts effects on the immune system by binding to the nuclear vitamin D receptor (VDR). VDR gene genetic variations are affecting serum vitamin D levels with a possible role in the BA risk. The current study aimed to examine the complex interaction of various factors (genetic background, serum vitamin D levels, and geographic location) to identify differences in the influence of these factors on the susceptibility to asthma between populations at different latitudes. Focusing on Eastern European cohorts from Latvia and Lithuania and comparing them with published data on East Asian populations, we explore the impact of VDR gene polymorphisms on BA susceptibility. Genotyping four key *VDR* SNPs and assessing their association with 25-hydroxyvitamin D levels, our study unveils significant associations of the studied loci with the risk of asthma—both risk-reducing and increasing effects, differently distributed between Baltic and East Asian populations. The functional effects of in silico VDR gene genetic variations are also identified and discussed.

## 1. Introduction

Asthma currently affects approximately 300 million people worldwide, and it is predicted that by 2025, an additional 100 million people may be affected [1,2,3], making this disease a major global health problem [4,5]. The pathogenesis of bronchial asthma (BA) involves a complex network of interacting factors at different disease levels and scales, from genetic to cellular to tissue and organ levels. These interactions are further complicated by environmental influences and non-asthma-related factors in patients, such as co-morbidities and lifestyle characteristics [6].

Vitamin D has immunomodulatory properties, as evidenced by the association of its deficiency with immune-mediated diseases, such as BA, rhinitis, and atopic dermatitis [7,8]. Research suggests that it plays a protective role against allergic diseases by promoting immune tolerance to allergens and reduces immunoglobulin E (IgE) sensitization, an important aspect in the pathogenesis of BA and other allergic diseases [9]. Therefore, vitamin D deficiency may be responsible for the increasing prevalence of allergic diseases and asthma worldwide. The human ability to produce the physiologically required amount of vitamin D under sun exposure decreases with increasing latitude. Recent studies have reported both positive [10,11] and negative associations [12] between the prevalence of asthma/allergies and latitude. The specific molecular mechanisms by which vitamin D can influence the course of BA are still unknown, but at the level of strong associations, vitamin D deficiency is associated with BA and asthma-related phenotypes [13,14]. Research investigating the impact of vitamin D status on asthma development focuses on prenatal maternal levels and their potential influence on newborns’ asthma symptoms [15,16]. Vitamin D deficiency may contribute to asthma by promoting early allergic sensitization in childhood linked to an increased risk of asthma, eczema, and sensitization [17]. Reduced vitamin D levels in asthmatic patients correlate with poorer responses to glucocorticoid therapy [15,18]. Long-term low vitamin D levels are associated with increased risks of inflammatory lung diseases and impaired lung functions [19], but monthly vitamin D supplementation has beneficial effects on lung functions, particularly in patients with asthma and chronic obstructive pulmonary disease [20]. Calcitriol (1,25(OH)2D3) impacts various cell types in asthma pathogenesis, reducing cell proliferation, inflammatory cytokines, and mucus secretion in airway muscle tissue models [21]. It also inhibits differentiation, migration, and cytokine production in non-specific immune cells involved in asthma [22].

Genetic factors seem to be more important in influencing the development of asthma, while lifestyle and environmental factors may play a more dominant role in the current manifestations of asthma [3]. It was also noted that risk factors should be analyzed in combination with other risk factors to account for potential interactions associated with this pathology. Numerous genome-wide association studies (GWASs) have shown a substantial genomic contribution to the etiology of asthma, with heritability estimates varying between 35% and 95% [23,24,25].

The VDR is a ligand-dependent transcription factor (TF) belonging to the nuclear receptor subgroup [26]. This receptor ensures that vitamin D functions in the cell by binding the hormonally active form of vitamin D or calcitriol (1,25(OH)2D). The complex of the VDR and 1,25(OH)2D further forms a heterodimeric structure with the retinoid X receptor (RXR), which can directly bind to vitamin D response element (VDRE) DNA regions in the gene promoter, as a transcription factor. Depending on the recruitment of other nuclear costimulatory or co-inhibitory factors, this complex can act as a ligand-dependent stimulator or inhibitor of gene transcription [27]. Point mutations of the VDR-coding gene could influence the function of this receptor; thus, these polymorphisms could cause susceptibility to various allergic diseases, including BA.

According to the Ensembl database (https://www.ensembl.org, accessed on 12 December 2023) data on SNPs of the VDR gene, a set of 15,174 SNPs have been identified, mostly covering the intronic or non-coding regions of the gene (14,426 SNPs). Of the polymorphisms identified in the coding part of the gene, 180 are synonymous and 365 are non-synonymous. The most studied polymorphisms in the VDR gene include rs2228570 (FokI) C>T, rs1544410 (BsmI) G>A, rs7975232 (ApaI) T>G, and rs731236 (TaqI) T>C. These polymorphisms are alternatively named after the corresponding restriction enzymes used in the analysis of restriction fragment length polymorphisms [28]. The VDR gene polymorphism rs2228570 is located in the start codon region, promoting structural changes in the protein, while rs1544410, rs7975232, and rs731236 are located in the 3′ regulatory region of the gene which has significance in mRNA stability and the recruitment of gene regulatory factors [29].

**Figure 1:**
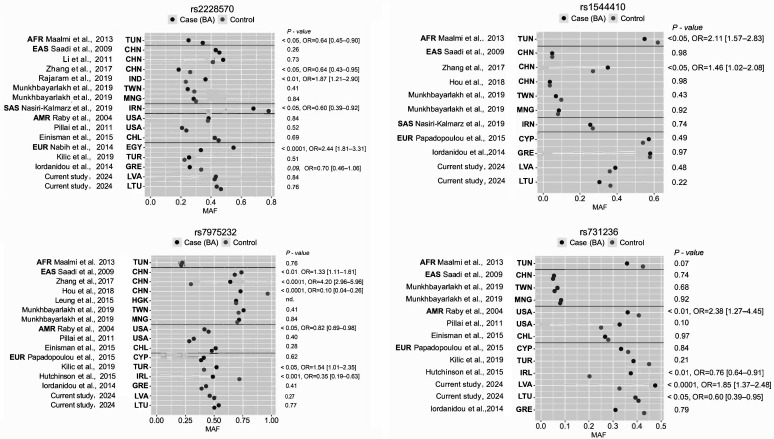
Associations of VDR gene SNPs rs2228570, rs1544410, rs7975232, and rs731236 with particular BA effects in different ethnic groups: minor allele frequency (MAF) distribution among controls and cases in population cohorts. Superpopulation: EAS (East Asia); AFR (African); AMR (Mixed American); EUR (Europe): CEU; and SAS (South Asia). ISO codes of countries are used according to the international standard ISO 3166 (https://www.iban.com/country-codes) [48]: Tunisia (TUN), China (CHN), Hong Kong (HGK), Taiwan (TWN), Mongolia (MNG), the United States of America (USA), Chile (CHL), Cyprus (CYP), Turkey (TUR), Ireland (IRL), Greece (GRE), Latvia (LVA), and Lithuania (LTU). MAF frequencies in the control group and disease cohort are indicated by light grey and dark grey circles, respectively; the *p*-*value* and OR of the association are given/calculated following the data from the publications [14,28,33,34,35,36,37,38,39,40,41,42,43,44,45,46,47].

During the last few years, various publications have described possible associations between VDR gene polymorphisms and the risk of autoimmune diseases. According to our meta-analysis, related to the PubMed database (https://pubmed.ncbi.nlm.nih.gov/), more than 110 publications have been published, investigating the relationship between VDR genetic variations, and rheumatoid arthritis, systemic lupus erythematosus, and multiple sclerosis [30,31,32]. The growing number of studies on the VDR gene rs2228570 (FokI), rs1544410 (BsmI), rs7975232 (ApaI), and rs731236 (TaqI) polymorphisms underscores the importance of understanding how genetic variation affects bronchial asthma susceptibility and progression. The existence of differences in the frequencies of the above SNPs’ genetic variations at racial and interpopulation levels has been reported in multiple case/control studies of asthma [14,28,33,34,35,36,37,38,39,40,41,42,43,44,45,46,47] (Figure 1).

The difference in minor allele frequencies between populations can lead to varying associations between genetic markers and bronchial asthma, as the prevalence of specific alleles may differ across populations due to historical, demographic, and evolutionary factors. These population-specific differences can impact the statistical power and effect sizes observed in genetic studies, highlighting the importance of considering diverse populations for a comprehensive understanding of the genetic basis of bronchial asthma. Therefore, the objective of our study was to uncover the interactions of genetic and environmental factors that contribute to geographic differences in asthma prevalence. To address this issue, measurements of serum 25(OH)D, genotyping of the VDR gene genetic variations in a case/control study, as well as a functional analysis in silico were carried out among populations of the Baltic region of Eastern Europe (Latvia and Lithuania); the results were subsequently compared with published data from a population study in East Asia to identify differences and/or common factors between geographically separated populations.

## 2. Results

### 2.1. Polymorphism Detection and Genetic Diversity

**Figure 2:**
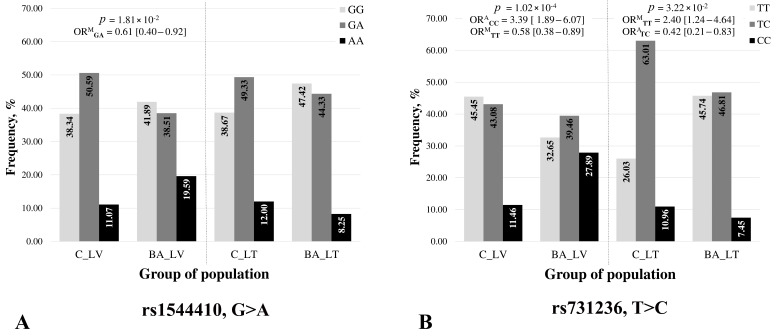
Comparison of the frequencies of genotypes: (**A**): rs1544410 (BsmI/GG, GA, and AA) and (**B**): rs731236 locus (TaqI/TT, TC, and CC) in the control (C) and bronchial asthma (BA) groups in Latvian (LV) and Lithuanian (LT) populations; probability (*p*) calculated by χ^2^ or Fisher exact test, and OR_A_, OR_M_—odds ratio according to the additive and multiplicative models with 95% confidence interval.

The data on the SNPs’ allele and genotype distributions in the Latvian (LV) and Lithuanian (LT) populations are presented in Tables S1 and S2 and Figure 2. The data from the Taiwanese association study were accordingly extracted for the comparative analysis [14]. In the Baltic population cohorts, all the SNPs studied were found to be in the HWE (*p* > 0.05). An association analysis with the disease FokI and ApaI polymorphisms showed a neutral effect in the LV and LT cohorts. Additionally, the heterozygote GA/rs1544410 (BsmI) manifested a slight protective effect only in Latvian people (*p* < 0.05; OR = 0.61 and 95% CI [0.40–0.92], respectively, Figure 2A).

In the current study, rs731236 (TaqI) was found to be significantly associated with BA in both populations and showed opposing risk-reducing and risk-increasing effects (Figure 2B). In the LV population, the rare allele C and the homozygote CC were found to be significantly more prevalent in the disease group relative to the controls and were identified as significant risk-increasing factors (*p* < 0.0001; OR_A_ = 1.85 and 95% CI [1.37–2.48] and OR_A_ = 3.39 and 95% CI [1.89–6.07]); the homozygotes and heterozygotes with the common allele T included showed a significant protective effect (*p* < 0.05; OR_A_ = 1.72 and 95% CI [1.12–2.63] and *p* < 0.001; OR_A_ = 1.27 and 95% CI [0.80–2.03]), respectively (Table S1 and Figure 2B). In the LT population, the rare allele C and heterozygote TC were the most represented in the controls and showed a minimal protective effect (*p* < 0.05) according to the additive model; the homozygote TT with common alleles included was found in significant association with the disease (*p* < 0.05; OR_M_ = 2.40 and 95% CI [1.24–4.64]) (Table S2 and Figure 2B).

### 2.2. Analysis of Serum 25(OH)D Levels in Case/Control Study among the LT and LV Populations

The results of a comparative analysis of the 25(OH)D levels in the case and control groups among the LV and LT populations are shown in Table 1.

A comparison of the BA groups revealed that the mean plasma 25(OH)D level was found to be sufficient in 68% of LV and 31.63% of LT patients; deficient in 26.39% of LV and 44.90%, it was critically low in 5.56% of LV and 23.47% of LT diseases cohorts.

Among the healthy individuals, the 25(OH)D level was sufficient in 77.55% of LV and 64.94% of LT controls; deficient in 20.41% of LV and 33.77% of LT; and critically low only in one representative of each population.

When analyzing the mean 25(OH)D level in the LV case/control study, no statistically significant difference was revealed between the patients with BA and the control group, as well as among the distribution of the 25(OH)D levels by groups: pronounced deficiency < 12 ng/mL; deficiency < 20 ng/mL; and sufficiency (>20 ng/mL) [49]. However, in the LV diseases cohort in the 25(OH)D sufficiency group, its level turned out to be higher than in the controls (32.10 versus 27.30 ng/mL, *p* = 4.43 × 10^−3^, Mann–Whitney test).

As a result of the comparison between the LV and LT disease cohorts, the mean 25(OH)D) levels were found to be statistically significantly different ((26.69 ± 10.74 ng/mL and 17.29 ± 6.87 ng/mL, respectively, *p* = 3.14 × 10^−9^) and among the BA groups: pronounced deficiency, deficiency, and sufficiency, *p* = 6.38 × 10^−6^).

When comparing the mean 25(OH)D) value among the controls of both populations, no significant difference was found (LV = 24.68 ± 7.68 ng/mL and LT = 24.24 ± 7.19 ng/mL); this result was also observed in the groups of healthy individuals stratified by the deficiency and sufficiency groups (*p* < 0.26).

Additionally, in the LT population, the mean 25(OH)D level was found to be statistically significantly elevated in the controls compared to the disease cohort (24.24 versus 17.29 ng/mL, *p* < 0.0001); it was significantly different between the groups stratified by pronounced deficiency, deficiency, and sufficiency (*p* < 0.0001, Table 1).

### 2.3. An Analysis of the Genetic Effect of the VDR Gene SNPs on Changes in the Serum 25(OH)D Levels in an Association Study

To reveal possible correlations between the mean serum 25(OH)D levels and specific VDR gene SNP genotypes, an association analysis of the above-mentioned parameters was performed in the case/control study (LT and LV, Tables S3 and S4). No statistically significant difference was found in the distribution of the mean 25(OH)D level between the genotypes of the rs2286570 (FokI), rs7975232 (ApaI), and rs731236 (TaqI). In the case of the polymorphism rs1544410 (BsmI), in the Latvian disease cohort, a homozygote AA for the rare allele was found in a correlation bordering on a statistically significant value (*p* = 0.052, *η* = 0.29) with an increased level of 25(OH)D (mean 32.47 ± 13.23 ng/mL), relative to other genotype variations (GG/mean, 23.88 ± 10.37 ng/mL, and GA/27.87 ± 9.23 ng/mL).

### 2.4. Identification of the Risk/Protective Multi-Loci Genotypes in Association with Serum 25(OH)D Level in the LT and LV Populations

**Figure 3:**
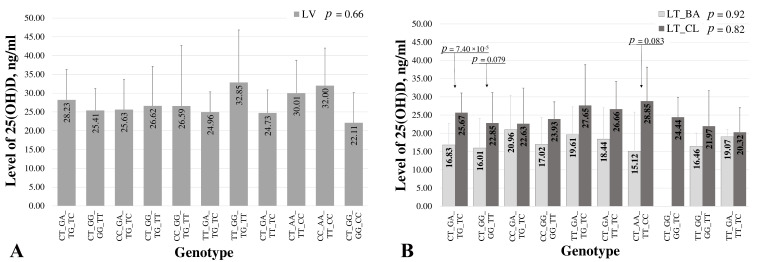
Distribution of risk/protective multi-locus genotypes in control (CL) and bronchial asthma (BA) groups according to mean 25(OH)D levels in serum in (**A**) Latvian (LV) and (**B**) Lithuanian (LT) populations.

The four multi-locus genotypes (rs2286570 (FokI)_rs1544410 (BsmI)_rs7975232 (ApaI)_rs731236 (TaqI)) distribution in each of our populations are shown in Figure 3.

Considering the fact that no statistically significant difference was found between the mean serum 25(OH)D level between the BA and a control group of healthy individuals in the Latvian population (Table 1), both groups were combined into one population cohort to examine the correlation between the VDR gene SNPs’ multi-locus genotypes composed and the mean serum 25(OH)D level. A statistical analysis did not show significant correlations in the general population of Latvia (Figure 3A). However, the highest mean serum 25(OH)D level (32.85 ± 13.97 ng/mL) was found for the genotype TT_GG_TG_TT with the homozygote TT of the rs731236 locus (TaqI) included, for which a protective effect against BA (*p* < 0.05) was previously established, and the lowest 25(OH)D level (22.11 ± 8.04 ng/mL), for the CT_GG_GG_CC genotype, including the rs731236 (TaqI) homozygote CC, was associated with a high BA risk (*p* < 0.0001) in the LV cohort, respectively.

In the LT population, a statistically significant difference was found between the mean 25(OH)D level in the control group relative to the disease cohort (*p* < 0.0001, Table 1). Thus, our task was to consider possible correlations of multi-locus genotype constructs between these cohorts regarding the mean level of 25(OH)D (Figure 3B). When considering each LT group separately, in the BA cohort, the mean 25(OH)D levels do not statistically significantly differ between carriers of all the variants of the multi-locus genotypes; the highest mean serum 25(OH)D level (20.96 ± 9.45 ng/mL) was found for the genotype CC_GA_TG_TC with the heterozygote TC (rs731236) included, which was previously shown to be a potentially protective factor for BA (*p* < 0.05, Table S2), and the lowest 25(OH)D level was for the genotype CT_AA_TT_CC (15.12 ± 10.68 ng/mL), respectively. Interestingly, in the control group, carriers of the CT_AA_TT_CC genotype had the highest mean level of 25(OH)D in the blood serum (28.85 ± 9.30 ng/mL), and the difference between the levels of the 25(OH)D carriers of this genotype in the control and group patients was on the verge of a statistically significant value (*p* = 0.083). The level of 25(OH)D in the carriers of another genotype CT_GG_GG_TT in a cohort of healthy individuals turned out to be prevalent and was found on the border of a statistically significant difference in comparison to representatives from the disease group (22.85 ± 8.33 ng/mL and 16.01 ± 8.07 ng/mL, respectively, *p* = 0.079). Healthy individuals, carriers of the genotype CT_GA_TG_TC, have a mean 25(OH)D level that is statistically significantly increased concerning carriers from the disease cohort (25.67 ± 5.39 ng/mL related to 16.8 ± 5.89 ng/mL, *p* < 0.0001, Figure 3B).

### 2.5. Eventual Functional Significance of the SNPs’ Allelic Variants

In the present study, we demonstrated the potential functional significance of rs731236 (TaqI) and rs1544410 (BsmI), which were previously identified in association with the BA and in correlation with the mean serum 25(OH)D levels, respectively.

**Figure 4:**
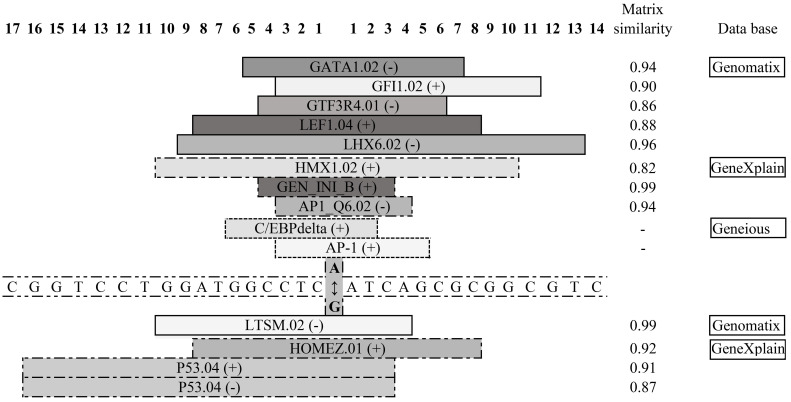
Consequences of the rs731236 (TaqI) A>G (chr12:47844974), according to position on chromosome, nucleotide substitutions on functional potential of corresponding genomic region. Data were obtained from Genomatix, Geneious, and GeneXplain databases. Localization of TFs on positive and negative DNA strands are indicated by (+) and (−), respectively. The TFs’ family and matrix names given according to MatInspector, Release 7.4 online tool at www.genomatix.de. GATA/GATA1.02—GATA-binding factor 1; GFI1/GFI1.02—Growth factor independence transcriptional repressor/Growth factor independence 1; GUCE/GTF3R4.01—GTF2IRDI upstream control element/GTF2I-like repeat 4 of GTF3; LEF1/TCF/LEF1.04—secondary DNA binding preference; LHXF/LHX6.02—Lim homeodomain factors/homeobox 6; LTSM/LTSM.02—Localized tandem sequence motif/LTSM elements with 6 bp spacer; /GEN_INI_B—general initiator seq. (viral+cellular); /C/EBPdelta-; /AP-1- and AP1_Q6_02—Activating Protein 1; /HOMEZ_01—Homeobox and leucine zipper protein Homez; and P53_04—tumor protein p53.

**Figure 5:**
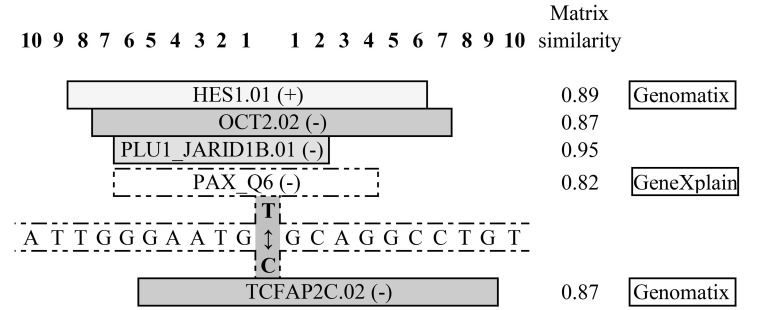
Consequences of the rs1544410 (BsmI), C>T (chr12:47846052), according to position on the chromosome, nucleotide substitutions on functional potential of a corresponding genomic region. Data were obtained from Genomatix and GeneXplain databases. Localization of TFs on positive and negative DNA strands are indicated by (+) and (−), respectively. The TFs’ family and matrix names are separated by the symbol of division and given according to MatInspector, Release 7.4 online tool at www.genomatix.de. V$HES1.01—Drosophila hairy and enhancer of split homologue 1 (HES-1); V$OCT2.02—Octamer-binding transcription factor-2, POU class 2 homeobox 2 (POU2F2); V$PLU1_JARID1B.01—Jumonji, AT-rich interactive domain 1B; V$PAX_Q6—TF in cellular protein metabolic process; M00808; V$TCFAP2C.02—Transcription factor AP-2, gamma.

Figure 4 and Figure 5 summarize the results of the in silico analysis of the functional significance of the rs731236 (TaqI) and rs1544410 (BsmI) allele substitutions evaluated on the eventual sequence affinity to transcription factors (TFs) and the splicing signals’ similarity.

In the case of the A>G substitution of the rs731236 locus, 10 TF binding sites are lost, and 4 new ones are created (Figure 4). A substitution for common allele A potentially assists in the creation of binding sites (BSs) in proteins of the GATA, GFI1, GUCE, LEF1, LHXF, and AP-1 families, but rare allele G assists in the creation of BSs to the TF of the LTSM, HONEZ, and p53 family.

In the case of the C>T substitution of the rs1544410 locus, a substitution for the rare allele T potentially assists in the creation of binding sites (BSs) in proteins of the HES, OCT, PLU, and PAX families, but common allele C assists in the creation of BSs to the TF of the TCFAP family (Figure 5).

**Figure 6:**
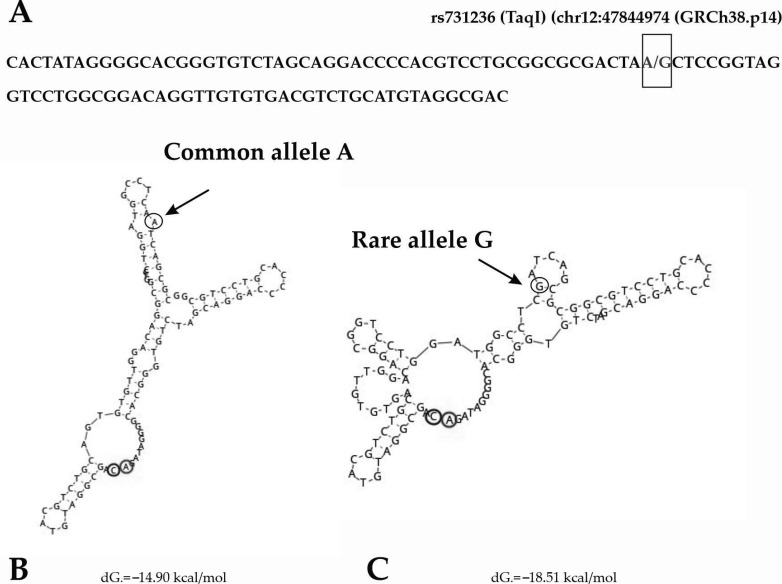
Impact of the SNPs rs731236 (TaqI) on the DNA secondary structure. (**A**) The sequence included nucleotide variations A>G (chr12:47844974) according to position on the chromosome, respectively. SNPs are indicated by an arrow and boxed; DNA secondary structures for (**B**) common and (**C**) rare alleles. Allelic substitutions are indicated by arrows, and free energy of optimal: dG.

The impact of the rs731236 (TaqI) on the DNA secondary structure was revealed; the nucleotide transition from A to G could generate the changes in the hairpin structure that could finally lead to thermodynamically more stable DNA secondary structures (Figure 6).

### 2.6. Effects of Polymorphism Sequence Changes on DNA Bends and Overall DNA Structure

**Figure 7:**
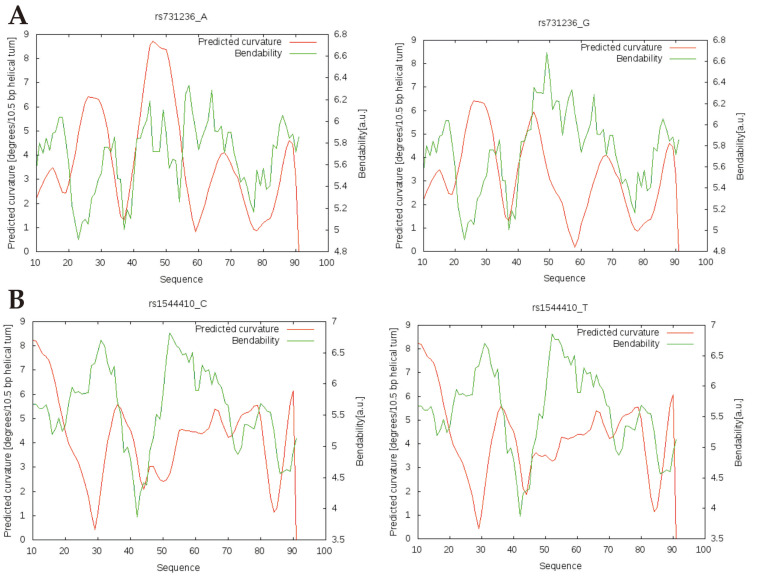
Possible changes in DNA curvature depend on the alleles of the sequence variants rs731236 and rs1544410, according to position on chromosome A>G (chr12:47844974) and C>T (chr12:47846052), respectively. (**A**): SNP rs731236 alleles. (**B**): SNP rs1544410 alleles. The red curve represents the predicted curvature of the DNA in degrees per 10.5 bp helical turn, and the green is the bendability. Both SNPs have position 51 in the analyzed sequence.

To determine the possible changes in the sequence and overall DNA structure that could presumably be affected by an allelic substitution of the investigated polymorphisms, characteristic graphs were constructed using Bend.it. and Model.it bioinformatics models (Figure 7).

As a result of a nucleotide substitution (G to A) of the rs731236, an increase in the DNA bending angle and a decrease in the bending ability can be observed. This change affects the sequence region from bp 42 to 55. In the case of allele A, the determined bending angle is 8.4°, and in the case of allele G, it is 2.7° (Figure 7A).

Alternative alleles of the rs1544410 do not change the degree of bending ability of the DNA sequence, but the C allele was found to reduce the bending angle of DNA by less than a degree (Figure 7B).

## 3. Discussion

The objective of this study was to examine the complex interaction of the *VDR* genetic background and serum vitamin D levels in Eastern European populations (Latvia and Lithuania) and to compare the data received with previously published data from East Asian populations (Taiwan and Mongolia), with the subsequent aim of identifying differences in the influence of these factors on the susceptibility to asthma between populations at different latitudes.

According to our research plan, we identified single-nucleotide polymorphisms of the VDR gene FokI (rs2228570), Bmsl (rs1544410), ApaI (rs7975232), and TaqI (rs731236) in case/control and functional in silico studies in Latvian and Lithuanian populations on the risk of bronchial asthma and compared the results obtained between two geographically distant regions located at different latitudes—the Baltic and Asian countries.

In both Baltic population cohorts, the FokI and ApaI polymorphisms showed a neutral effect (Tables S1 and S2). Consistent with Munkhbayarlakh et al.’s 2019 published data [14], ApaI (rs7975232) was also not found in association with the disease in the Taiwanese and Mongolian groups; however, the *VDR* FokI (rs2228570) GG genotype played significant roles in terms of conferring the risk of asthma and showed marginal significance in the Taiwan group (*p* = 0.065). It was found to be more prevalent in the Mongolian (41.1%) than in the Taiwanese group (27.0%) but did not show any association with asthma in Mongolia.

The recessive model of the rs2228570 GG genotype (vs. AG+AA) has also been found to be associated with the risk of developing asthma (*p* < 0.02) in Taiwanese people [14]. In turn, this genotype was found to be equally distributed in both Baltic populations between the controls and cases (among the Latvians, 27.70% in cases and 30.83% in controls, and among the Lithuanians, 30.39% in cases and 26.67% in controls) and showed a neutral effect in association with disease. The existence of differences in the frequencies of the above SNPs’ genetic variations at racial and interpopulation levels has been reported. Zhao et al. [50] identified that ApaI (rs7975232) is implicated in childhood asthma among Asian people, the FokI polymorphism is associated with childhood asthma in Caucasian people, and the BsmI polymorphism does not significantly contribute to asthma susceptibility in children; a recent report also suggested an association between the TaqI and ApaI polymorphisms and asthma in Irish children [28].

The distribution of the Bmsl (rs1544410) and TaqI (rs731236) genotypes in the populations of Taiwan and Mongolia turned out to be identical between populations, with a significant predominance of homozygotes for common alleles (more than 80% in each population) [14]. In the Baltic countries, TaqI (rs731236) was found to be significantly associated with the risk of asthma—both risk-reducing and increasing effects, differently distributed among Latvia and Lithuania. In LV, the rare allele C and the homozygote CC were identified as significant risk-increasing factors (*p* < 0.0001). Additionally, the homozygotes and heterozygotes with the common allele T included showed a significant protective effect (*p* < 0.05 and *p* < 0.001, respectively (Figure 2B)). In the LT population, in turn, the rare allele C and heterozygote TC showed a minimal protective effect, but the homozygote TT with common alleles included was found to have a significant association with the disease (*p* < 0.05; Figure 2B). Additionally, the heterozygote GA/rs1544410 (BsmI) showed a slight protective effect only in Latvians. These findings underscore the complexity of genetic influences on asthma susceptibility, with variations influenced by both ethnic and geographic factors in Eastern European and Asian populations.

In recent years, it has been suggested that vitamin D deficiency is associated with asthma, which may explain much of the increase in the incidence of this disease in children [7]. In a comparative analysis of the vitamin D levels in the populations of two geographically located countries of the Baltic region, Latvia, and Lithuania, we unexpectedly discovered a significant difference both between the general population experimental groups and between the disease cohorts and healthy individuals in each population.

No difference in the mean 25(OH)D levels as well as among its distribution in the BA and controls stratified by the deficiency and sufficiency groups was found in Latvia (Table 1). Interestingly, in the cohort of LV patients with BA in the group of sufficient 25(OH)D (>20 ng/mL), its level was found to be higher than in the analogic LV control group (32.10 versus 27.30 ng/mL, *p* < 0.01). Because the plasma samples were collected for our experiment at the same time of year (September–April) from individuals not taking vitamin D supplements, we can rule out these conditions potentially influencing the experiment and infer the existence of any additional mechanisms and genetic, epigenetic, and environmental factors influencing the maintenance of the balance of this important vitamin in patients with asthma in the LV population. Diet was not considered in our study but may have been critical in maintaining vitamin D balance in this case. Various case–control studies have found differences in vitamin D levels between patients with asthma and healthy controls, particularly decreased vitamin D levels in a cohort of patients with asthma [51,52]. In the LT population, the mean 25(OH)D level was significantly elevated in the controls compared to the patients with BA group (*p* < 0.0001) and differed between the groups stratified by deficiency and sufficiency (*p* < 0.0001). Among the healthy individuals, it was similar in LV (24.68 ± 7.68 ng/mL) and LT (24.24 ± 7.19 ng/mL) and was sufficient in 77.55% of LV and 64.94% of LT controls. In the LT disease group, the 25(OH)D level was significantly lower than for LV patients (26.69 ± 10.74 ng/mL and 17.29 ± 6.87 ng/mL, respectively, *p* < 0.0001), with sufficiency in 68% of LV and 31.63% of LT patients. Additionally, a critically low 25(OH)D level was observed in 5.56% of the LV and 23.47% of LT disease cohorts (Table 1). The difference in the mean 25(OH)D levels in the asthma cohort between Latvia and Lithuania may be due to factors such as genetics, diet, and lifestyle that may also contribute to this difference by affecting the overall availability and absorption of vitamin D. Additional research that takes these factors into account may help to understand more in detail the mechanisms behind the observed differences.

As it was reported [14], in Asian populations, specifically in Taiwan and Mongolia, a notable disparity in the serum vitamin D concentrations was also observed between asthmatic and non-asthmatic individuals. The study revealed that the non-asthmatic Taiwanese cohort exhibited significantly higher vitamin D levels compared to both patients with asthma in Taiwan and individuals, both asthmatic and non-asthmatic, in Mongolia across various age groups. There is high consumption of seafood, milk, and mushrooms in Taiwan, which are the main sources of vitamin D food and can have some effect on the results [14,53]. Furthermore, within both populations, children with asthma consistently exhibited significantly lower vitamin D levels compared to their non-asthmatic cohort, underscoring a potential association between asthma and lower vitamin D levels in Taiwan and Mongolia. Moreover, the majority of Mongolian cohorts, whether asthmatic or non-asthmatic, exhibited vitamin D concentrations below 20 ng/mL. Additionally, a vitamin D blood concentration below the threshold of 40 ng/mL was associated with a significantly elevated risk of asthma (*p* < 0.0001) in Taiwan.

It is believed that individual genetic differences may be responsible for differences in vitamin D levels [54]. Among these potential genetic determinants, genetic variations in the VDR gene have been considered in different populations [55]. A statistical analysis did not reveal any significant differences in the mean vitamin D levels among genotypes of the investigated SNPs rs2286570 (FokI), rs1544410 (BsmI), rs7975232 (ApaI), and rs731236 (TaqI) among the Baltic populations. However, in the Latvian diseases cohort, a potential association and protective effect was noted for the rs1544410 (BsmI) polymorphism, where individuals with the homozygote AA genotype for the rare allele exhibited a correlation bordering on statistical significance (*p* = 0.052) with an elevated level of mean 25(OH)D (32.47 ± 13.23 ng/mL) compared to other genotype variations (GG/23.88 ± 10.37 ng/mL and GA/27.87 ± 9.23 ng/mL). As reported by Munkhbayarlakh et al., 2019 [14], in the Taiwanese population, the combination of the VDR polymorphism rs2228570 with a serum vitamin D concentration below 40 ng/mL, particularly in individuals with the GG genotype with common alleles included, conferred the highest risk of bronchial asthma. Additionally, according to the above-mentioned study [14], the Mongolian population is likely to be more susceptible to generalized vitamin D deficiency, regardless of individual *VDR* genetic variants, and therefore has a greater risk of susceptibility to allergic diseases and asthma. Overall, mixed evidence suggests that vitamin D has protective properties in the context of asthma, but the precise nature of this interaction is unclear, as evidenced by inconsistent and conflicting data from studies in this area [56].

To test the possible complex effect on the level of vitamin D in bronchial asthma of the genetic background of the VDR gene, we conducted a correlation analysis of multi-locus genotypes in the cohorts of disease and healthy individuals in the Baltic populations and also revealed significant differences between countries. Among the Latvian population, there were no notable variations in the average serum 25(OH)D levels between individuals with BA and the control group (Figure 3A). However, a significant difference in the mean 25(OH)D levels emerged between the control and disease cohorts in Lithuania, prompting an in-depth examination of the multi-locus genotype correlations in each LT group. Within the LT bronchial asthma cohort, carriers of the CC_GA_TG_TC genotype (with one locus genotype TC previously identified as a protective factor for BA) included exhibited the highest mean 25(OH)D levels, while carriers of the CT_AA_TT_CC genotype had the lowest levels. Notably, in the control group, carriers of the CT_AA_TT_CC genotype had the highest mean 25(OH)D levels, bordering on statistical significance compared to the patients. (Figure 3B). These findings suggest a potential association between specific genotypes and vitamin D levels in Lithuania, warranting further exploration of the genetic and vitamin D interplay in bronchial asthma.

In silico predictions were employed in the current study to identify allele-specific patterns of transcription factor binding sites (TFBSs) for SNPs rs1544410 (BsmI) and rs731236 (TaqI), previously found in association with BA or vitamin D levels in the present study. The obtained results show that the investigated allelic variations affect TF binding—in the case of rs731236 A>G (chr12:47844974), 10 TF binding sites are lost and 4 new ones are created, (Figure 4), while in the case of the rs1544410 C>T (chr12:47846052), allele T creates binding links for 4 new TFs; the common allele C assists in the creation of BSs only for 1 TF. These changes in the recruitment of transcription factors are potentially able to significantly modulate the transcription of the VDR gene and in the case of certain diseases are directly related to the disease phenotype [57]. Asthma is characterized by inflammation, marked by the elevated transcription of pro-inflammatory proteins, including cytokines and enzymes. Many transcription factors are common to several cell types (ubiquitous) and may play a general role in the regulation of inflammatory genes, whereas others are cell-specific and may determine the phenotypic characteristics of a cell. Families of transcription factors, such as AP-1 and GATA regulating inflammatory gene expression, play a pivotal role in asthma’s pathogenesis, amplifying and perpetuating inflammation, thereby impacting disease severity and treatment responsiveness [58].

In the case of the allelic substitution of the sequence variant rs731236 (TaqI), probable changes in the secondary structure (SS) of DNA were also detected. The DNA SSs are formed when the DNA double helix unwinds during replication and transcription. These detected changes in the secondary structure of DNA can potentially affect the interaction of a given sequence region with various regulatory proteins [59], thereby potentially impacting processes, such as DNA replication, transcription, and DNA error repair [60].

The results reveal that both allelic variants of rs731236 impact DNA bending, a crucial factor in gene expression regulation and DNA packaging, and replication, linked to the binding capacity of regulatory proteins [61]. Specifically, the rs731236 nucleotide change (A>G) decreases the DNA bending angle and increases the bending capacity, suggesting challenges in recruiting sequence-specific regulatory proteins to the specific DNA region [62].

Many studies highlight the beneficial functional properties of vitamin D in the context of asthma. Meta-analysis shows that serum vitamin D levels are positively correlated with improvements in lung function in patients with asthma, which is explained by the anti-inflammatory properties of vitamin D in the lungs—1,25(OH)2D3 reduces the production of inflammatory cytokines (such as IL-8) and chemokines (such as CXCL10, which promotes leukocyte recruitment) from stimulated lung epithelial cells [56]. Also, the hormonally active form of vitamin D can activate macrophages, stimulate the production of Th1 (first type T helper cells) cytokines, as well as inhibit Th2-related asthma inflammation, which is the basis of asthma pathogenesis. Th2 cell-associated cytokines (e.g., IL-4, IL-5, and IL-13) are important in the development of allergic responses by activating IgE-secreting B cells [63]. Vitamin D also influences eosinophil activity by reducing IgE synthesis and increasing the expression of the anti-inflammatory IL-10 [64]. Since vitamin D performs its functions by binding to the VDR, point mutations in its encoding gene may affect the function of this receptor; therefore, these polymorphisms may cause predisposition to various allergic diseases, including asthma. It was also concluded [14] that in the subtropical region of Taiwan, serum vitamin D concentrations and VDR gene variants were found to be significant risk factors for the development of asthma, whereas, in the temperate highland region of Mongolia, vitamin D levels stood out as a major determinant of asthma risk. Our ongoing Baltic region study suggests that, concerning asthma, both the genetic component and vitamin D function as independent factors influencing risk or protection. This underscores the complexity of asthma predisposition. In summary, our studies further highlight the relationship between genetics, vitamin D, environmental factors, and asthma. Lifestyle, diet, and other environmental influences also play a role in the development and manifestation of asthma.

Calcitriol metabolism is controlled by a complex interaction of genetic, dietary, and environmental factors, making it difficult to obtain standardized data on vitamin D levels. This might partly explain the lack of associations in our study. Although serum samples were collected to determine vitamin D from study participants over a short period, this did not exclude seasonal variations in weather, the amount of melanin pigment in the skin, and lifestyle factors.

## 4. Materials and Methods

### 4.1. Case–Control Study

A total of 149 (54 female) and 98 patients (66 female) represented the Latvian (LV) and Lithuanian (LT) asthma groups, respectively (Table S5). The LV patients with BA were enrolled in the outpatient clinic of Riga Bikernieki Hospital, Latvia. The LT study was carried out at the Hospital of Lithuanian University of Health Sciences Kauno Klinikos.

The diagnosis of bronchial asthma for the LV and LT patients was established according to Global Initiative of Asthma criteria (GINA; http://www.ginasthma.org/local/uploads/files/GINA_Under5_Pocket_20091_1.pdf, accessed on 17 December 2023). The patients in both populations had mild-to-moderate asthma with no exacerbation during the past year. The treatment of patients included the inhalation of short- and long-acting adrenergic receptor agonists in combination with corticosteroids. At the time of this study, the patients had forced expiratory volume in the first second (FEV1)/forced vital capacity (FVC) > 85% and FEV1 > 80% of predicted values, based on demographic characteristics. The asthma control test showed well-controlled asthma >20.

Blood samples for the LT and LV studies were collected during the autumn–winter period from patients with asthma and vitamin D supplement-free healthy individuals, excluding any other conditions that could adversely affect the study results.

The LV population was represented by 252 controls (59% were females); mean age: 48.52 ± 9.07 years) and was obtained based on the Latvian Center for Marine Medicine, Vecmilgravis Hospital (15 DNA samples), and Riga Bikernieki Hospital, which specialized in trauma medicine (44 DNA samples). A total of 193 DNA samples for the control group were referred to the Genome Database of Latvian Population, Latvian Biomedical Research and Study Center (http://biomed.lu.lv/gene/). Individuals without autoimmune and cardiovascular disorders, type 2 diabetes mellitus (T2DM), and obesity were included in this cohort. All the subjects are a mixture of representatives of non-Baltic ethnic groups of Riga, forming some “average” genotype for North-Eastern Europe.

The LT population was represented by 77 healthy individuals (49 females; mean age: 35.08 ± 12.41 years), who underwent prophylactic evaluation at Kaunas Family Medicine Centres and Hospital of Lithuanian University of Health Sciences and were without diagnosis or familial predisposition to congenital diseases; acute or chronic infections; oncological, autoimmune, or any other chronic diseases, as well as immunodeficiency; and obesity.

The studies in the LV and LT populations were performed according to the Declaration of Helsinki, and both study protocols were approved by the Central Medical Ethics Committee of Latvia (IRB No: 01-29.1.2/4798) and Lithuania (IRB No: BE-2-74), respectively. Informed consent was obtained from all participants of this study.

### 4.2. DNA Extraction and Genotyping

Genomic DNA for the LV study was extracted from nucleated blood cells using a kit for genomic DNA extraction (Thermo Fisher Scientific, Vilnius, Lithuania). The VDR gene polymorphisms (FokI (rs2228570), TaqI (rs731236), BsmI (rs1544410), and ApaI (rs7975232)) were genotyped via restriction fragment length polymorphism (RFLP) analysis. DNA for the LT study was isolated using the QIAamp DNA blood mini kit (Qiagen, Hilden, Germany) according to the manufacturer’s instructions. The VDR gene polymorphisms (rs7975232, rs1544410, rs731236, and rs2228570) were analyzed using TaqMan SNP Genotyping Assays probes were sourced from Thermo Fisher Scientific (https://www.thermofisher.com), according to the manufacturer’s protocol. The genotyping data were verified via direct sequencing of the corresponding DNA fragments in both directions using the Applied Biosystems 3130*xl* Genetic Analyzer, (Thermo Fisher Scientific, Waltham, MA, USA). The loci description and nucleotide numbering is given according to the recommended of Human Genome Variation Society (HGVS) nomenclature system (https://varnomen.hgvs.org/). The chromosome 12 GRCh38.p14 assembly (NCBI reference sequence: NC_000012.12) sequence information was used for the loci description.

### 4.3. Measurements of Serum 25(OH)D in Blood Serum

The measurements of serum 25(OH)D were performed in LV patients with BA (*n* = 72) and control group (*n* = 48) individuals, using enzyme-linked immunoassay (25-OH-Vitamin D ELISA, IBL International GmbH, TECAN), and in LT patients with BA (*n* = 98) and control group (*n* = 77) individuals, using the DIAsource 25OH vitamin D Total ELISA kit (Louvain-la Neuve, Belgium), respectively. The analysis kit detection limit was defined as the apparent concentration of two standard deviations below the average OD at zero binding, which was 1.5 ng/mL. The vitamin D levels were stratified according to the serum 25(OH)D concentrations, pronounced deficiency < 12 ng/mL and deficiency < 20 ng/mL and above 20 ng/mL, according to the European Tissue Society (ECTS) guidelines, the International Society of Endocrinology of the United States Institute of Medicine, and the Scientific Advisory Committee on Nutrition [49].

### 4.4. Data Management, Sample Size, and Statistical Analysis

The sample size was estimated according to the data on asthma incidences in the LV and LT populations [65,66], using a standardized sample size calculation formula (the number of required study subjects is 30–35 samples). Single-locus genotypes and allele frequencies were estimated by direct gene counting. These primary genotyping data were used to construct multi-locus genotypes and identify the main effects of BA in single- and multi-locus models among populations. The differences between the case and control groups in the allele and genotype frequencies were evaluated via the Fisher exact test using IBM SPSS Statistic v.25 (IBM Corp. Released 2017). Genetic models for the investigated locus were designed using different contingency tables and their relationships to the underlying genetic model. An odds ratio (OR) of more than two (2) and less than 0.5 was clinically significant [67]. Differences in the serum 25(OH)D measurements between the case/control and cohort studies were determined using an appropriate statistical method, considering the number of comparison groups and the normality of the data within them. The method was chosen from *t*-tests, ANOVA, Mann–Whitney, and Kruskal–Wallis tests. The association between the group assignment and 25(OH)D level was determined using the eta (*η*) value. For all the statistical analyses, statistical significance was assumed at *p* < 0.05.

### 4.5. SNP Functional Analysis In Silico

An eventual functional significance of the SNPs showing evidence of association was analyzed in silico on the sequence similarity to the transcription factor binding sites (TFBSs) related to the position on chromosome GRCh38.p14 and using the Genomatix software, Version 8.6, MatInspector, Release 7.4 online tool [68]; TRANSFAC in GeneExplaine online tool (https://genexplain.com/transfac/); and TRANSFAC in Geneious Prime^®^ 2021.0.3 (Classification of Human Transcription Factors (Draft 5 October 2018, http://www.edgar-wingender.de/huTF_classification.html). Only parameters with a core/matrix similarity of 1.00/0.85 or more were considered. The DNA secondary structures were predicted using the Mfold web server [69] (http://www.unafold.org/). Folding was simulated at 37 °C and with 20 mM Na^+^ and 1.5 mM Mg^++^ for Intracellular or/and 145 mM Na^+^ and 0.5 mM Mg^++^ for Extracellular [70]. If various similar structures were obtained, the structures with the highest negative free energy were representative. To analyze the effect of allelic substitution on the bending ability of the DNA molecule, the Bend.it server was used (http://pongor.itk.ppke.hu/dna/bend_it.html). Possible bends of DNA based on the dinucleotide were performed according to the geometric properties of the angles (tilt and rotation angles). The vector sums were calculated using Godsell and Dickerson’s BEND algorithm [71] and expressed in degrees per 10.5 helical turns [72].

## 5. Conclusions

We suggest that patterns of *VDR* gene structural variations may reflect an ethnic group’s historical or geographic adaptation processes, influencing present-day human health. Our study highlights regional variations in asthma risk, particularly in the Baltic region, emphasizing the equal importance of genetic components and vitamin D status as significant determinants while recognizing the intricate interplay of genetics, vitamin D, and environmental factors in asthma susceptibility.

## Figures and Tables

**Table 1:** Comparative analysis of 25(OH)D levels in case and control groups among LV and LT populations.

25(OH)D, ng/mL	Distribution and the mean value of 25(OH)D				
	Frequency, %	Mean ± SD [95% CI]		Frequency, %	Mean ± SD [95% CI]
	BA _LV (*n* = 72)			BA _LT (*n* = 98)	
Level in plasma	-	26.69 ± 10.74 [24.17–29.21]		-	17.29 ± 6.87 [15.91–18.67]
<12 ng/mL	05.56	09.19 ± 1.99 [6.02–12.35]		23.47	09.71 ± 2.40 [8.67–10.75]
<20 ng/mL	26.39	16.41 ± 1.80 [15.55–17.28]		44.90	15.28 ± 2.04 [14.66–15.90]
≥20 ng/mL	68.06	32.10 ± 8.48 [29.67–34.54]		31.63	25.78 ± 4.25 [24.22–27.34]
25(OH)D	C_ LV (*n* = 49)			C_LT (*n* = 77)	
Level in plasma	-	24.68 ± 7.68 [22.48–26.89]		-	24.24 ± 7.19 [22.61–25.88]
<12 ng/mL	2.04	3.69 (1 sample)		1.30	10.66 (1 sample)
<20 ng/mL	20.41	16.83 ± 2.64 [14.94–18.72]		33.77	16.56 ± 2.25 [15.65–17.46]
≥20 ng/mL	77.55	27.30 ± 6.26 [25.25–29.36]		64.94	28.51 ± 4.90 [27.12–29.91]
Associations (*p* value) analysis among the groups					
		C _LV vs. C_LT	BA _LV vs. C_LV	BA_LT vs. BA _LV	BA_LT vs. C_LT
Distribution in all groups		n.s.	n.s.		1.00 × 10^−6^
Mean level in plasma:					
In all samples		n.s.	n.s.	3.14 × 10^−9^	2.38 × 10^−9^
In samples with <20 ng/mL		n.s.	n.s.	6.38 × 10^−6^	2.41 × 10^−2^
In samples with ≥20 ng/mL		n.s.	4.43 × 10^−3^		9.88 × 10^−3^

Control (C) and bronchial asthma (BA) groups in Latvia (LV) and Lithuania (LT) populations; *n*—sample number, SD—standard deviation, CI 95%—95% confidence interval, and n.s.—no significant association.

## Data Availability

The data that support the findings of this study are available from the corresponding author upon reasonable request.

## Supplementary Materials

The following supporting information can be downloaded at: https://www.mdpi.com/article/10.3390/ijms25031943/s1.
