# Supplementary material for: Genetic Diversity in Bronchial Asthma Susceptibility: Exploring the Role of Vitamin D Receptor Gene Polymorphisms in Varied Geographic Contexts
Source: Int J Mol Sci. 2024 Feb 5;25(3):1943. doi: 10.3390/ijms25031943 (PMC10856277; doi:10.3390/ijms25031943)
Supplement: Supplementary file 1 [file ijms-25-01943-s001.zip › ijms-2805209-supplementary.pdf]

Supporting information

Table S1. Comparison of MAF and genotype frequencies of VDR gene loci in Bronchial asthma and controls in Latvian population.

Table S2. Comparison of MAF and genotype frequencies of VDR gene loci in Bronchial asthma and controls in Lithuania population.

Table S3. An association analysis between the average 25(OH)D level of genotypes of VDR gene SNPs in Bronchial asthma and controls in Latvian population.

Table S4. An association analysis between the average 25(OH)D level of genotypes of VDR gene SNPs in Bronchial asthma and controls in Lithuanian population.

Table S5. Demographic characteristics of study subjects

Table S1. Comparison of MAF and genotype frequencies of VDR gene loci in Bronchial asthma and Control groups in Latvian (LV) population.

SNPs of the VDR gene	MAF or genotype	Distribution of alleles and genotypes				Statistical analysis			
		BA patients		Control group		Additive model		Multiplicative model	
		n	%	*n*	%	OR_A [95% CI]	*p*	OR_M [95% CI]	*p*
	T	128	43.24	215	42.49	1.03 [0.77 - 1.38]	0.84	-	-
rs2286570	CC	41	27.70	78	30.83	1.16 [0.74 - 1.82]	0.51	0.86 [0.55 - 1.35]	0.51
C>T	CT	86	58.11	135	53.36	1.21 [0.76 - 1.93]	0.65	1.21 [0.81 - 1.83]	0.36
	TT	21	14.19	40	15.81	1.00 [0.52 - 1.91]	1.00	0.88 [0.50 - 1.36]	0.66
	A	115	38.85	184	36.36	1.11 [0.83 - 1.49]	0.48	-	-
rs1544410	GG	62	41.89	97	38.34	0.86 [0.57 - 1.30]	0.48	1.16 [0.77 - 1.75]	0.48
G>A	GA	57	38.51	128	50.59	0.70 [0.45 - 1.09]	0.11	0.61 [0.40 - 0.92]	1.92×10^{-2}
	AA	29	19.59	28	11.07	1.62 [0.88 - 2.98]	0.12	1.04 [0.60 - 1.80]	0.90
	T	136	46.26	253	50.00	1.18 [0.88 - 1.57]	0.27	-	-
rs7975232	GG	34	23.13	57	22.53	0.98 [0.60 - 1.58]	0.91	1.03 [0.63 - 1.66]	0.92
T>G	GT	68	46.26	139	54.94	0.82 [0.49 - 1.37]	0.13	0.70 [0.46 - 1.05]	0.08
	TT	46	31.29	57	22.53	1.35 [0.76 - 2.41]	0.30	0.81 [0.52 - 1.25]	0.33
	C	140	47.62	167	33.00	1.85 [1.37 - 2.48]	4.16×10^{-5}	-	-
rs731236	TT	48	32.65	115	45.45	1.72 [1.12 - 2.63]	1.20×10^{-2}	0.58 [0.38 - 0.89]	1.20×10^{-2}
T>C	TC	58	39.46	109	43.08	1.27 [0.80 - 2.03]	1.02×10^{-4}	0.86 [0.57 - 1.30]	0.48
	CC	41	27.89	29	11.46	3.39 [1.89 - 6.07]	2.73×10^{-5}	1.41 [0.85 - 2.36]	0.18

n – number of patients in Control and bronchial asthma (BA) groups; *p-values* by $\chi 2$ test; OR_A OR_M – Odds ratio by additive or multiplicative model; 95% CI – confidence interval, statistically significant *p-values* ($p < 0.05$) in bold.

Table S2. Comparison of MAF and genotype frequencies of VDR gene loci in Bronchial asthma and Control groups in Lithuanian (LT) population.

SNPs of the VDR gene	MAF or genotype	Distribution of alleles and genotypes				Statistical analysis			
		BA patients		Control group		Additive model		Multiplicative model	
		n	%	*n*	%	OR_A [95% CI]	*p*	OR_M [95% CI]	*p*
rs2286570 C>T	T	85	43.81	70	46.67	0.94 [0.61 - 1.43]	0.76	-	-
	CC	30	30.39	20	26.67	0.78 [0.40 - 1.53]	0.47	1.28 [0.65 - 2.49]	0.47
	CT	49	50.52	44	58.67	0.74 [0.37 - 1.49]	0.40	0.77 [0.42 - 1.40]	0.38
	TT	18	18.56	13	17.33	0.92 [0.37 - 2.29]	0.86	1.38 [0.63 - 3.06]	0.42
rs1544410 G>A	A	59	30.41	55	36.67	0.75 [0.48 - 1.19]	0.22	-	-
	GG	46	47.42	29	38.67	0.70 [0.38 - 1.29]	0.25	1.43 [0.78 - 2.64]	0.25
	GA	43	44.33	37	49.33	0.73 [0.39 - 1.39]	0.34	0.82 [0.32 - 2.44]	0.82
	AA	8	8.25	9	12.00	0.56 [0.19 - 1.62]	0.28	0.89 [0.32 - 2.44]	0.82
rs7975232 T>G	T	96	51.06	73	50.00	0.94 [0.62 - 1.43]	0.77	-	-
	GG	27	28.72	23	31.51	0.96 [0.48 - 1.91]	0.90	1.05 [0.52 - 2.08]	0.90
	GT	46	48.94	35	47.95	1.00 [0.48 - 2.10]	1.00	1.17 [0.60 - 2.30]	0.64
	TT	25	26.60	19	26.03	0.89 [0.39 - 2.02]	0.78	1.17 [0.60 - 2.30]	0.64
rs731236 T>C	C	58	30.83	62	42.43	0.60 [0.39 - 0.95]	2.82×10^{-2}	-	-
	TT	43	45.74	19	26.03	0.42 [0.22 - 0.81]	0.06	2.40 [1.24 - 4.64]	8.90×10^{-3}
	TC	44	46.81	46	63.01	0.42 [0.21 - 0.83]	1.22×10^{-2}	0.52 [0.28- 0.96]	0.04
	CC	7	7.45	8	10.96	0.39 [1.12 - 1.22]	0.10	0.88 [0.30 - 2.55]	0.81

n – number of patients in Control and bronchial asthma (BA) groups; *p-values* by $\chi 2$ test; OR_A OR_M – Odds ratio by additive or multiplicative model; 95% CI – confidence interval, statistically significant *p-values* ($p < 0.05$) in bold.

Table S3. An association analysis between the average 25(OH)D level of genotypes of VDR gene SNPs in Bronchial asthma and Control groups in Latvian population.

Locus / variation	Group (*n*)	Genotype	*n*	25(OH)D) level, ng/ml								*p* / *η*
				Mean	SD	Min	Max	CI95%		Median	IQR	
rs2228570 C>T	BA (72)	TT	12	28.05	14.58	6.39	56.56	18.79	37.31	24.87	18.66	0.86 / 0.08
		CT	38	26.08	9.91	9.60	48.06	22.82	29.33	24.93	17.23	
		CC	22	27.01	10.18	9.66	45.19	22.49	31.52	26.49	17.68	
	Control (47)	TT	12	26.53	5.55	20.93	37.52	23.00	30.05	24.79	8.00	0.68 / 0.13
		CT	24	24.05	7.71	3.63	39.34	20.79	27.30	23.97	8.33	
		CC	11	24.78	10.18	13.83	49.69	17.94	31.62	23.41	13.21	
rs1544410 G>A	BA (72)	AA	11	32.47	13.23	9.60	48.06	23.58	41.36	38.91	19.96	0.056 / 0.29
		GA	27	27.87	9.23	14.79	45.19	24.21	31.52	25.97	16.25	
		GG	34	23.88	10.37	6.39	56.56	20.27	27.50	24.15	13.56	
	Control (47)	AA	11	24.47	8.74	3.63	37.52	18.59	30.34	24.96	7.73	0.98 / 0.03
		GA	15	24.77	6.78	13.01	34.95	21.02	28.53	23.81	9.97	
		GG	21	25.11	8.31	14.53	49.69	21.33	28.89	24.12	8.29	
rs7975232 T>G	BA (72)	GG	18	24.44	7.02	13.36	38.73	20.95	27.93	25.15	11.44	0.58 / 0.13
		TG	35	27.15	11.51	6.39	56.56	23.19	31.10	23.86	16.88	
		TT	19	27.97	12.28	9.60	48.06	22.06	33.89	25.97	24.86	
	Control (47)	GG	12	24.71	6.42	13.01	39.34	20.63	28.79	23.81	7.19	0.73 / 0.12
		TG	25	24.23	8.88	3.63	49.69	20.56	27.89	23.81	9.90	
		TT	10	26.59	6.70	19.33	37.52	21.80	31.38	25.47	13.50	
rs731236 T>C	BA (72)	CC	34	26.88	10.83	6.39	47.90	23.10	30.66	25.04	21.52	0.21 / 0.21
		TC	26	24.42	9.13	9.66	44.01	20.74	28.11	23.11	15.32	
		TT	12	31.06	13.03	9.60	56.56	22.78	39.34	27.72	17.09	
	Control (47)	CC	7	26.70	6.72	19.33	37.52	20.49	32.91	24.96	12.47	0.13 / 0.12
		TC	15	23.79	7.82	3.63	33.95	19.46	28.12	23.81	9.53	
		TT	25	24.97	8.22	13.01	49.69	21.58	28.36	24.12	8.29	

n – number of patients in groups; SD - standard deviation; CI 95% - 95% confidence interval of mean; IQR – inter quarter interval; *p* – Statistical significance of mean with ANOVA; *η* – a measure of association.

Table S4. An association analysis between the average 25(OH)D level of genotypes of VDR gene SNPs in Bronchial asthma and Control groups in Lithuanian population.

Locus / variation	Group (*n*)	Genotype	*n*	25(OH)D) level, ng/ml								*p* / *η*
				Mean	SD	Min	Max	CI95%		Median	IQR	
rs2228570 C>T	BA (97)	TT	30	18.75	7.22	10.06	33.28	16.06	21.45	17.20	11.60	0.28 / 0.16 KW
		CT	49	16.33	6.99	2.03	33.53	14.32	18.34	14.85	8.23	
		CC	18	17.96	5.59	8.22	28.68	15.17	20.74	17.48	10.10	
	Control (77)	TT	20	22.11	6.76	10.66	35.57	18.95	25.28	20.55	12.39	0.27 / 0.19 A
		CT	44	25.25	6.93	12.64	42.24	23.14	27.35	26.61	9.55	
		CC	13	24.14	8.51	14.23	36.63	19.00	29.28	22.51	17.70	
rs1544410 G>A	BA (97)	AA	8	19.35	7.30	12.84	33.53	13.25	25.45	15.94	10.90	0.50 / 0.13 KW
		GA	43	17.84	7.19	5.69	33.47	15.62	20.05	15.59	10.49	
		GG	46	16.56	6.54	2.03	30.02	14.62	18.50	15.33	9.80	
	Control (75)	AA	9	26.74	7.54	18.23	38.34	20.95	32.54	27.54	14.21	0.38 / 0.16 A
		GA	37	24.71	7.45	10.66	42.24	22.23	27.20	26.41	10.94	
		GG	29	23.08	6.94	12.64	36.71	20.43	25.72	24.06	12.13	
rs7975232 T>G	BA (98)	GG	25	17.59	8.01	5.69	33.53	14.28	20.89	14.85	8.43	0.32 / 0.13 KW
		TG	46	17.97	6.17	6.23	32.51	16.14	19.80	16.61	9.53	
		TT	27	15.87	6.94	2.03	29.97	13.12	18.61	13.87	10.78	
	Control (77)	GG	19	24.69	6.99	14.23	42.24	21.32	28.06	23.34	9.08	0.62 / 0.11 A
		TG	35	24.82	7.77	10.66	38.34	22.15	27.49	26.90	12.39	
		TT	23	23.00	6.58	12.64	36.71	20.15	25.84	24.06	11.75	
rs731236 T>C	BA (94)	CC	7	19.95	7.67	12.84	33.53	12.85	27.04	16.70	12.67	0.25 / 0.18 KW
		TC	44	18.29	7.19	5.69	33.47	16.11	20.48	16.98	10.37	
		TT	43	16.25	6.47	2.03	30.02	14.26	18.24	14.85	8.56	
	Control (73)	CC	8	25.96	7.66	18.23	38.34	19.56	32.37	24.02	13.68	0.40 / 0.10 A
		TC	46	24.90	7.28	10.66	42.24	22.73	27.06	26.61	10.23	
		TT	19	22.50	7.42	12.64	36.71	18.92	26.07	24.06	12.67	

n – number of patients in groups; SD - standard deviation; CI 95% - 95% confidence interval of mean; IQR – inter quarter interval; *p* – Statistical significance of mean with ANOVA (A) or Kruskal-Wallis (KW) test; *η* – a measure of association.

Table S5. Demographic characteristics of study subjects

Population	N (%) / Mean (SD)		*p*
	**BA patients**	**Control group**	
**Latvian** (*n* = 401)	149	252	
Sex (female)	54 (56%)	148 (59%)	<0.0001
Age	11.72 (9.77)	48.52 (9.07)	<0.0001
25(OH)D level in plasma	26.69 (10.74)	24.68 (7.68)	n.s.
**Lithuania** (n = 175)	98	77	
Sex (female)	66 (67%)	49 (64%)	0.072
Age	42.68 (13.56)	35.08 (12.41)	<0.0001
25(OH)D level in plasma	17.29 (6.87)	24.24 (7.19)	<0.0001

n – number of patients in Control (C) and bronchial asthma (BA) groups; *p* – Statistical significance of mean with ANOVA
